# Effects of land cover and air pollution on the risk of preterm births

**DOI:** 10.11606/s1518-8787.2024058005504

**Published:** 2024-03-04

**Authors:** Tiana C. L. Moreira, Jefferson L. Polizel, Weeberb J. Réquia, Paulo Hilario Nascimento Saldiva, Demostenes F. da Silva Filho, Silvia Regina Dias Medici Saldiva, Thais Mauad

**Affiliations:** I Universidade de São Paulo Faculdade de Medicina Departamento de Patologia São Paulo SP Brazil Universidade de São Paulo. Faculdade de Medicina. Departamento de Patologia. São Paulo, SP, Brazil; II Escola Superior de Agricultura “Luiz de Queiroz” Departamento de Ciências Florestais Piracicaba SP Brazil Escola Superior de Agricultura “Luiz de Queiroz”. Departamento de Ciências Florestais. Piracicaba, SP, Brazil; III Fundação Getúlio Vargas Escola de Políticas Públicas e Governo Brasília DF Brazil Fundação Getúlio Vargas. Escola de Políticas Públicas e Governo. Brasília, DF, Brazil; IV Universidade de São Paulo Faculdade de Medicina Departamento de Obstetrícia e Ginecologia São Paulo SP Brazil Universidade de São Paulo. Faculdade de Medicina. Departamento de Obstetrícia e Ginecologia. São Paulo, SP, Brazil

**Keywords:** Parks, Recreational, Air Pollution, Infant, Premature, Built Environment

## Abstract

**OBJECTIVE:**

To evaluate the association between gestational age and green areas, urban built areas, and the concentration of particulate matter 2.5 (PM_2.5_) in the city of São Paulo, analyzing the irregular distribution of these areas and pollution levels above the recommended level.

**METHODS:**

The study population consisted of a cohort of live births from 2012, and data from the Live Birth Information System (Sinasc) of the city of São Paulo were used. Using satellite images and supervised classification, the distribution and quantity of green areas and built areas in the city of São Paulo was obtained, as well as the concentrations of PM_2.5_. Logistic regressions were used to obtain possible associations.

**RESULTS:**

The results of the study show that a lower percentage of green areas is significantly associated with a higher chance of preterm births. A higher building density was positively associated with the odds ratio for preterm birth. We did not find any significant associations between air pollution (PM_2.5_) and preterm births.

**CONCLUSIONS:**

The results of this study show that greener areas are less associated with preterm births when compared with less green areas.

## INTRODUCTION

The association of gestational outcomes with environmental exposures, namely urban green areas and air pollution, is an important field of study of environmental epidemiology.

Several studies show that living near green areas and frequenting them brings benefits regarding birth weight and/or prematurity^1^. Grazuleviciene et al.^4^ carried out a study with 3,416 women in the first trimester of pregnancy in Lithuania, investigating the beneficial influence of a shorter distance between mothers’ homes and public parks on maternal systemic blood pressure. In another study, pregnant women who lived up to 1,250 m (a 10 to 15 minutes’ walk) from green areas in cities in Pennsylvania (USA) were found to have a higher frequency of full-term pregnancies than those who lived further than 1,250 m from green areas^5^.

Groups with lower purchasing power seem to benefit especially from exposure to green areas. In China, mothers with lower purchasing power exposed to these areas obtained the greatest benefits in terms of birth outcomes, especially those related to prematurity^6^. In Australia, Akaraci et al.^1^ observed that a greater coverage of green areas was related to lower chances of prematurity in the more socially vulnerable population, those with lower purchasing power.

Other studies found no evidence that living near green or less polluted areas reduced the risk of preterm birth. Asta et al.^7^, for example, observed that women in Rome, even those who lived closer to green areas, had an increased probability of preterm birth with each 1°C increase in temperature, with no modifying effect from particulate matter 10 (PM_10_).

Several studies worldwide found that maternal exposure to particulate matter causes adverse effects on prematurity and birth weight^8^. Confirming these data, a study carried out in the city of São Paulo found that an increase of 10 μg/m^3^ in O_3_ and PM_10_ was associated with chances of prematurity and low birth weight^9^.

In 2012 and 2013, 348,337 live births were recorded in the city of São Paulo, with a prematurity rate of 11.9%, which ranged from 8.4% to 15.9% in the 96 districts of the city of São Paulo^10^, a figure that is very similar to global averages^11^. Leal et al.^12^ observed that, in a population of 23,940 Brazilian women in 2011 and 2012, 11.5% of births were premature; and that adolescent mothers with low schooling and income were the majority in this rate.

**Figure:**
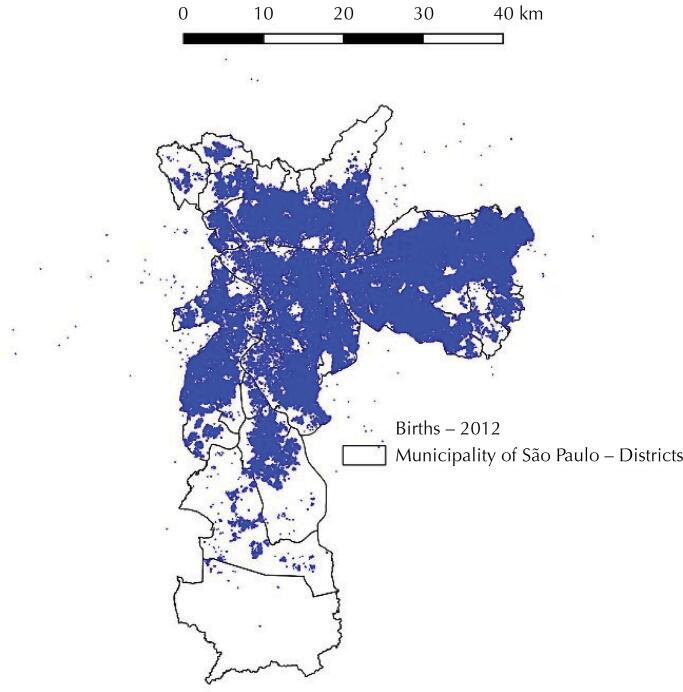
Study population in the municipality of São Paulo.

Few studies have analyzed environmental variables and their influence on gestational outcomes in São Paulo. Most of the studies focusing on this association were carried out in countries with higher purchasing power than ours^8 , 13 , 14^ or in cities where the characteristics of pollution and green area distribution differed from those of our megacities. In São Paulo, green areas are distributed quite unevenly and concentrated in wealthier, intra-urban locations. The green areas located in peripheral regions are remnants of the native ones, not located in the urban fabric, and have little recreational use^15^. The levels of PM_2.5_ in the city of São Paulo, measured by the stations of the Environmental Company of São Paulo State (Cetesb), exceed the maximum limits established by the World Health Organization (WHO)^18 , 19^.

In this study, we present data and analyze the exposure to green and built areas, maternal PM_2.5_, and odds ratio for preterm birth in the city of São Paulo in 2012.

## METHODS

### Study Area

The city of São Paulo has an area of 1,521 km^2^ and, in 2012 had a population of 11.37 million inhabitants^20^. The city’s climate, according to Koppen, is Cwa (humid subtropical climate), which is characterized by a dry winter and a rainy summer^21^.

The distribution of green areas in the city of São Paulo is uneven, as demonstrated by Amato et al.^17^. The largest concentrations of green areas are in regions of environmental protection, on the edges of the city.

### Study population

The database of the Live Birth Information System (Sinasc) for 2012, geocoded by the mothers’ full address, was provided by the Epidemiology and Information Coordination of the São Paulo Health Secretariat ( Figure ), in a partnership for the development of the research project on Prematurity in the Municipality of São Paulo, approved by the Ethics Committee of the Municipal Health Secretariat (CAEE 26132714.1.0000.0086). For this study, the mothers’ addresses were grouped at the level of city districts. SINASC provides variables such as birth weight, gestational age, type of pregnancy (single, double, triple), presence of congenital anomalies, type of delivery, age, education and occupation of the mother, and number of previous births (Ordinance SVS nº 116/2009, 11/02/2009).

In this study, 174,215 records of single live births were analyzed. The following variables were used for the analysis: birth weight, gestational age, type of delivery, date of birth, Apgar 5 scale, and mother’s age, marital status and schooling^22^. The database was processed by keeping only birth weight above 0.5 and below 5 kg and excluding rows with blank data.

The age of the pregnant woman was categorized into groups: under 15; between 15 and 19.9; between 20 and 34.9; between 35 and 39.9; and over 40.

The variable for maternal educational level (last grade completed) was used as it appears in the SINASC database (educational level), as described below:

0 – No schooling;

1 – Primary Education I (1st to 4th grade);

2 – Primary Education II (5th to 8th grade);

3 – Secondary Education (9th to 12th grade);

4 – Incomplete Higher Education;

5 – Complete Higher Education.

### Pregnancy Outcomes

This study used the gestational age records provided by the SINASC database, which were generated both based on the day of the last menstrual period and by ultrasound.

Gestational age was classified according to the Guidelines for Perinatal Care (American Academy of Pediatrics) and the American College of Obstetricians and Gynecologists^23^:

Extremely preterm: less than 28 weeks;

Very preterm: 28 weeks to 31 weeks and 6 days;

Moderate preterm: 32 weeks to 33 weeks and 6 days;

Late preterm: 34 weeks to 36 weeks and 6 days;

Preterm: less than 37 weeks;

Early term: 37 weeks to 38 weeks and 6 days;

Full term: 39 0/7 weeks to 40 weeks and 6 days;

Late term: 41 0/7 weeks to 41 weeks and 6 days;

Post-term: 42 weeks or more.

A binary variable (0,1) was created for the number of gestational weeks: preterm equals 1 (less than 37 weeks) and not preterm equals 0 (more than 37 weeks).

The variable “type of delivery” was divided into caesarean section or vaginal delivery.

The APGAR score is a system used to quickly assess the health of a newborn shortly after birth. It was developed by Dr. Virginia Apgar in 1952 and consists of five categories: heart rate, respiratory effort, muscle tone, irritability reflex, and skin color. It can be assessed in the first minute after birth (APGAR 1), five minutes after birth (APGAR 5) and, sometimes, 10 minutes after it, when the score is below 5. In this study, we will use the APGAR 5^24 , 25^.

### Assessment of Exposure and Land Cover

The area of green exposure and land cover was analyzed according to the municipality’s administrative division into 96 districts^26^.

GeoSES was used as a socio-environmental correction factor, summarizing the main dimensions of the Brazilian socioeconomic context for research purposes^27^. GeoSES is a composite index and its dimensions are education, mobility, poverty, social and material deprivation, income, wealth, and segregation.

Pollution data was obtained from satellite images taken by the Copernicus Atmosphere Monitoring Service (CAMS) in 2012. Particulate matter 2.5 (PM_2.5_) data in μg/m^3^ were extracted from the images, using the average for the period in each district of the city of São Paulo^28^. This study used the annual means of pollutant, which do not consider the variations over the year, such as the seasons and climatic conditions.

Using the QGIS2.18.11 program, two different indicators of exposure to green areas were used: vegetation cover and the number of street trees. A digital map of the locations of street trees in São Paulo in 2010 was provided by the municipality itself (Geosampa)^29^. The map includes urban trees on sidewalks, street islands and traffic circles, and excludes trees in squares, parks, reserves and indoor public and private areas. The images had a resolution of 2 m at a scale of 1:25,000.

The orthophotos used in this study were provided by the Institute of Geosciences and Cartography of the State of São Paulo and had a spatial resolution of 2 meter-pixel, with three spectral bands: near infrared (NIR), blue and red. Land cover was classified using the Random Forest (RF) algorithm (QGIS2.18.11 program; Dtezaka Plugin). RF is a powerful learning classification algorithm, as well as one of the most accurate methods for classifying land cover^30^. In addition, it is a general term for ensemble methods that use tree-structured classifiers to train the algorithm, which creates several trees similar to the Classification and Regression Tree (CART)^31^.

In the classification, for each tree in the RF, there is a vote for the most popular class (pixel color) in the data input (polygon training sample = data input). The output of the classifier is determined by the majority of votes in the class^32^. For the training samples, classes were classified according to pixel color and spectral signature. In total, 150 training samples were prepared for each Land cover class. The images were classified into the following Land cover classes: tree canopy, grass cover, bare soil, cement floor, swimming pool, shade, roof (white, gray, dark, ceramic), asphalt, and river/lake (adapted from Myeong et al.^33^, 2003). For data analysis, the sum of the tree canopies and grass coverwas considered green space and the sum of the different roof types was considered built environment.

A false-color composite scheme was used to enable the detection of vegetation in the image. In this type of representation, vegetation appears in different shades of red, depending on its type and condition, due to its high reflectance in the NIR band^34^. Bare soil, roads, and buildings can appear in various shades of blue, yellow, or gray, depending on the composition material. The false color orthophoto composition used in this study was R (channel 1) = NIR band, G (channel 2) = red band, B (channel 3) = blue band.

Classification accuracy was determined using a classification error matrix, the Kappa index. The thematic maps used in this study had Kappa values equal to or greater than 81%, which is considered an accurate classification according to Landis and Koch^35^.

Databases of street trees and Land cover were evaluated in the 96 districts.

### Data Analysis

These analyses were carried out to understand the association of the gestational week with environmental and sociodemographic exposure variables. Each variable was assessed in unadjusted models, which were then adjusted for logistic models in which the variables showed an association with at least one of the outcomes in the analysis. Binary logistic regression was used for the dichotomous dependent variables. The significance level adopted was p = 0.05. The results of the logistic regressions were presented as odds ratios (95% confidence intervals).

Logistic models were controlled for ethnicity, age, educational and marital status, air pollution, and socioeconomic status.

Land cover was used in quartiles and divided as follows ( Table 2 ):

**Table 2:** Distribution of quartiles of Land cover and particulate matter (PM)2,5.

Land cover	Q1 (%)	Q2 (%)	Q3 (%)	Q4 (%)
Green areas	15.4 (24.8)	25.8 (33.6)	34.6 (45.0)	46.0 (84.8)
Tree cover	6.9 (14.7)	15.7 (23.3)	24.3 (33.6)	34.6 (73.4)
Grass cover	3.4 (8.0)	9.05 (9.31)	10.31 (11.6)	12.6 (17.7)
Built area	4.6 (21.0)	22.0 (29.9)	30.9 (36.7)	37.7 (49.7)
PM_2.5_	11.8 (12.1)	13.2 (12.3)	13.3 (12.7)	13.7 (13.7)
Street trees	0.5 (4.8)	5.82 (6.8)	7.8 (9.73)	10.73 (16.2)

Q1 - the lowest percentage of Land cover within 25% of the population;

Q2 - the second lowest percentage of Land cover within 25% of the population (up to the median);

Q3 - the second highest percentage of Land cover within 25% of the population (above the median);

Q4 - the highest percentage of Land cover within 25% of the population.

To avoid multicollinearity, all models were built separately for each Land cover variable. A new variable called “green area” was created, defined as the sum of trees and grass cover; another variable called “built area” was established as the sum of the areas of all the roofs of different colors. The significance level adopted was p < 0.05.

## RESULTS

In total, 174,215 records of single live births were found; the cases in which addresses were outside the city of São Paulo and those that did not contain information on the variables used in the study were excluded. Thus, 166,384 records were used, as shown in Table 1 .

**Table 1:** Descriptive analysis of birth variables (1a - sociodemographic and 1b - newborn health).

Characteristics	n	%
Sociodemographic (n = 166,384)		
Maternal age		
Mean (CV%)	27.7 (23.8%)	
Median [Min, Max]	28.0 [11.0, 52.0]	
Standard deviation	6.6	
Marital status of the mother		
Single	75,261.	43.2
Married	68,082	39.08
Widow	278	0.16
Separated/divorced	2,451	1.41
Stable union	27,878	16
No data	58	0.03
Educational level		
No schooling	17	0.01
Primary Education I	294	0.17
Primary Education II	2,019	1.16
Secondary Education	20,487	11.76
Incomplete Higher Education	103,185	59.23
Complete Higher Education	47,896	27.49
No data	169	0.1
Newborn health (n = 166,384)		
APGAR Scale		
0		0.09
1		0.13
2		0.05
3		0.06
4		0.11
5		0.22
6		0.33
7		0.94
8		4.23
9		34.86
10		58.65
No data		0.1
Presence of congenital abnormalities		
Yes		1.84
No		98.02
No data		0.1
Gestational age		
Preterm	18,434	12.31
Early term	61,147	36.06
Full term	73,537	42.63
Late term	9,702	5.62
Post-term	3,628	2.12
Birth Weight (CV)		
Mean (CV)	3,162.90	17.7
Median [Min, Max]	[500, 4,990]	
Standard deviation	354	
No data	6	0
Gestational age at delivery (CV)		
Mean (CV%)	38.3	5.8
Median [Min, Max]	39.0 (19.0, 46.0)	
Standard deviation	2.21	
No data	2,196	1.3

Table 2 shows the distribution of quartiles according to Land cover, demonstrating that “green area” varies from 15.4% to 84.8%, while air pollution varies very little, from 11.8 to 13.7 μg/m^3^.

Table 3 shows the results of the “unadjusted and logistic models” analysis. The marital statuses “single” and “stable union” showed significant associations (increases of 2% and 4%, respectively) with preterm birth in relation to the marital status “married,” both in the logistic model analyses without adjustment and in the logistic analysis. The unadjusted logistic model analysis showed significance for all age groups, in relation to the 20 to 35 age group. In the logistic analysis, only age groups over 35 years were associated with preterm birth. The GeoSES mean was found to be positively associated with prematurity in the regressions of the unadjusted logistic model, although marginally, but this association did not occur in the logistic analysis.

**Table 3:** Results of unadjusted models and logistic models for gestational age at delivery < 37 weeks and Land cover.

Gestational Age at Delivery		No adjustment			Logistic		
	Predictor	Odds ratio	CI	p	Odds ratio	CI	p
Marital status - ref: single	Married ref	---------	------------	------	-------	-------------	------
	Single	1.11	1.08 – 1.15	**0.001**	1.12	1.04 – 1.12	**0.001**
	Widow	1.09	0.73 – 1.56	0.629	1.10	0.71 – 1.52	0.747
	Separated/divorced	1.06	0.92 – 1.20	0.379	1.06	0.92 – 1.20	0.418
	Stable union	1.11	1.06 – 1.16	**0.001**	1.11	1.02 – 1.12	**0.003**
Educational level - ref: no schooling	No schooling	1.07	0.63 – 1.80	0.794	1.06	0.60 – 1.73	0.815
	Primary Education I	1.46	1.04 – 2.05	**0.026**	1.42	1.00 – 1.97	**0.040**
	Primary Education II	1.27	1.10 – 1.45	**0.001**	1.23	1.07– 1.41	**0.003**
	Secondary Education	1.20	1.14 – 1.26	**0.001**	1.17	1.10 – 1.23	**0.001**
	Incomplete Higher Education	1.09	1.04 – 1.12	**0.001**	1.06	1.02 – 1.10	**0.003**
	Complete Higher Education ref	---------	------------	------	-------	-------------	------
Age - ref: 20-34.9	Ref: 20-34.9	---------	------------	------	-------	-------------	------
	= 15	1.59	1.30 – 1.92	**0.001**	1.08	0.86 – 1.36	0.472
	15-19.9	1.27	1.21 – 1.32	**0.001**	1.05	0.99 – 1.11	0.064
	35-39.9	1.19	1.13 – 1.23	**0.001**	1.20	1.14 – 1.27	**0.001**
	≥40	1.43	1.32 – 1.54	**0.001**	1.30	1.18 – 1.42	**0.001**
Ethnicity - ref: white	White	---------	------------	------	-------	-------------	------
	Black	1.09	1.02 – 1.15	**0.006**	1.04	‘	0.142
	Asian	0.93	0.80 – 1.06	0.278	0.94	0.82 – 1.08	0.426
	Mixed-race	1.01	0.97 – 1.04	0.519	0.96	0.93 – 1.00	0.065
	Indigenous	0.86	0.68 – 1.06	0.177	0.80	0.63 – 0.99	**0.050**
Type of delivery - ref: vaginal	Vaginal delivery	---------	------------	------	-------	-------------	------
	Cesarean section	0.98	0.94 – 1.00	0.145	0.96	0.92 – 0.99	**0.039**
Prenatal appointments	Number of appointments	0.933	0.92 - 0.93	**0.001**	0.93	0.93 - 0.94	**0.022**
Birth data	APGAR at 5 minutes	0.82	0.81 – 0.83	0.001	0.97	0.96 – 0.98	**0.001**
	Birth weight (kg)	1.00	0.99 – 0.99	**0.001**	0.99	0.99 – 0.99	**0.001**
Green spaces - quartiles	Green Area Q1 [15.4-24.8]	1.07	1.02 – 1.11	**0.002**	1.08	1.03 – 1.13	**0.001**
	Green Area Q2 [25.8-33.6]	1.04	0.99 – 1.08	0.104	1.05	1.00 – 1.10	**0.018**
	Green Area Q3 [34.6-45]	0.99	0.94 – 1.029	0.516	0.99	0.94 – 1.3	0.671
	Green Area Q4 [46-84.8]	---------	------------	------	-------	-------------	------
Tree cover - quartiles	Tree cover Q1 [6.9-14.7]	1.06	1.01 – 1.10	**0.011**	1.07	1.02- 1.12	**0.001**
	Tree cover Q2 [15.7-23.3]	1.03	0.98 – 1.07	0.206	1.04	0.99-1.09	0.059
	Tree cover Q3 [24.3-33.6]	0.98	0.93 – 1.02	0.384	0.98	0.94 – 1.03	0.571
	Tree cover Q4 [34.6-73.4]	-------	-------------	------	-------	-------------	------
Grass cover- quartiles	Grass cover - Q1 [3.4-8]	1.11	1.06 – 1.16	**0.001**	1.09	1.04 – 1.14	**0.001**
	Grass cover - Q2 [9.05-9.31]	1.11	1.06 – 1.16	**0.001**	1.08	1.03 – 1.13	**0.001**
	Grass cover - Q3 [10.31-11.6]	1.04	0.99 – 1.08	0.125	1.02	0.97 – 1.07	0.301
	Grass cover - Q4 [12.6-17.7]	-------	-------------	------	-------	-------------	------
Built area - quartiles	Built area - Q1 [4.6-21]	---------	------------	------	-------	-------------	------
	Built area - Q2 [22-29.9]	1.02	0.97 – 1.06	0.297	1.03	0.99 – 1.08	0.123
	Built area - Q3 [30.9-36.7]	1.02	0.97 – 1.06	0.314	1.02	0.97 – 1.07	0.357
	Built area - Q4 [37.7-49.7]	1.09	1.04 – 1.13	**0.001**	1.09	1.04 – 1.14	**0.001**
PM_2.5_ - quartiles	PM_2.5_ - Q1 [11.8-12.1]	---------	------------	------	-------	-------------	------
	PM_2.5_ - Q2 [13.2-12.3]	0.98	0.93 – 1.01	0.275	0.97	0.93 – 1.02	0.380
	PM_2.5_ - Q3 [13.3-12.7]	1.02	0.97 – 1.06	0.388	1.01	0.96 – 1.05	0.613
	PM_2.5_ - Q4 [13.7-13.7]	0.99	0.94 – 1.03	0.546	1.00	0.95 – 1.05	0.943
	GeoSES Mean	0.96	0.91 – 0.99	**0.035**	1.00	0.95 – 1.05	0.915
Street trees – quartiles (ref: 9.73-16.2)	Street trees - Q1 [0.53-4.82]	1.01	0.96 – 1.05	0.595	1.00	0.95-1.04	0.940
	Street trees - Q2 [5.82-6.8]	0.97	0.92 – 1.00	0.11	0.95	0.91-0.99	**0.034**
	Street trees - Q3 [7.8-9.73]	1.00	0.95 – 1.04	0.975	1.01	0.95-1.04	0.945
	Street trees - Q4 [9.73-16.2]	-------	-------------	------	-------	-------------	------

PM: particulate matter; 95%CI: 95% confidence interval.

In the logistic regression analysis ( Table 3 ), prematurity was associated with the marital statuses “single” (OR = 1.12; 95%CI 1.04-1.12; p = 0.001) and “stable union” (OR = 1.1; 95%CI 1.02-1.12; p = 0.003) in relation to marital status “married”; the presence of street trees within the different districts in the second quartile (Q2: OR = 0.95; 95%CI 0.91-0.99; p = 0.034), in relation to the fourth quartile, was associated with a greater chance of preterm birth. Mothers aged over 35 years, from 35 to 39.9 years (OR = 1.20; 95%CI 1.14–1.27; p = 0.001) and over 40 years (OR = 1.30; 95%CI 1.18–1.4; p = 0.001) were found to have a higher chance of giving a premature birth when compared to the mothers aged from 20 to 34.9 years. The percentage of built areas within each district in the fourth quartile (OR = 1.09; 95%CI 1.04-1.14; p = 0.001) showed an increased association with prematurity in relation to the first quartile. The total percentage of green areas (Q1: OR = 1.08; 95%CI 1.03–1.13; p = 0.001 and Q2: OR = 1.05; 95%CI 1.00–1.10; p = 0.018), tree cover (Q1: OR = 1.07; 95%CI 1.02–1.12; p = 0.001), and grass cover (Q1: OR = 1.09; 95%CI 1.04–1.14; p = 0.001 and Q2: OR = 0.91; 95%CI 1.00–1.10; p = 0.018) showed an association with prematurity and higher chances of preterm birth in relation to the fourth quartile of each of the respective land covers. The number of prenatal appointments was also associated with preterm birth.

No statistical associations were found between the concentration of PM_2.5_ in the districts and the number of preterm births.

## DISCUSSION

In this study, we analyzed the influence of different types of Land cover on the frequency of preterm births in the city of São Paulo in 2012. We found that the percentage of different types of green areas in the city’s districts reduced the chance of preterm birth by 5% to 9%. The results of this study also showed that a lower percentage of green areas is significantly associated with a higher chance of prematurity, as are densely built areas (> 37.7% of built area, in this study). The PM_2.5_ levels did not influence the parameters studied. To our knowledge, this is the first study to show beneficial associations between urban green areas and reduced prematurity in Brazil.

Prematurity was also found to be related to other factors, such as ethnicity, marital status, maternal age, type of delivery, prenatal appointments, birth weight, and Apgar score, which has already been confirmed in the literature^36^. In this study, we observed that Indigenous people have a higher chance of giving a premature birth, as described by Martinelli et al.^36^ and Barreto et al.^37^.

The mother’s marital status and age also influence prematurity. In this study, pregnant women over the age of 35, single or in a stable union were found to be more likely to give birth prematurely, and these findings corroborate the results of other studies^38^.

An increased number of prenatal appointments is also well established in the literature as a factor that promotes a lower risk of prematurity^36 , 39^, which was also observed in this study.

Many studies have shown the beneficial association between exposure to green areas and pregnancy outcomes, but most of them were carried out in countries with a higher income or Human Development Index. Meta-analyses revealed that these associations are maintained when there is a high percentage of residential green space.

Villeuneuve et al.^41^ found that the percentage of residential green space was positively associated with a reduction in the risk of preterm birth, low birth weight and small-for-gestational-age birth, unlike this study, in which the only factor found to be positively associated with residential green area was prematurity. Studies associating socioeconomic status with greater benefits from green areas indicate that these beneficial effects are greater in disadvantaged populations^42^, with the exception of an Australian study that showed disproportionate benefits among women from more affluent neighborhoods^43^. In this study, we used the GeoSES index within each district, but significant associations between this index and prematurity in the different districts were not found in the logistic analysis. It was discovered that, in the city of São Paulo, a greater number of green areas is not necessarily linked to socio-economic indices. In the southern part of the city, for example, there are large areas of native vegetation cover in districts with low socio-economic indices, and in the western region, the highest levels of vegetation cover are in the more affluent areas of the city—although the quality of and access to these green areas are probably different in the two regions.

The specific mechanisms by which green areas benefit pregnancy are unknown, but there are some possibilities: these areas may cause 1) a restorative psychological effect on mothers, by reducing stress; 2) a direct effect on cardiovascular health, by increasing physical activity, and on mental health, by stimulating social cohesion; 3) an indirect effect on cardiovascular health, by improving environmental conditions of pollution, temperature, and humidity^43^.

Green areas can improve the microclimate of regions and reduce pollution^2^. However, as Akaraci et al.^40^ in a study in Sydney, Australia, we found no association between PM_2.5_ and prematurity, unlike studies in other countries^40 , 43 , 44^. Because the pollution measurements used in this study were derived from satellite images, individual exposure differences may not have been captured. Furthermore, this result points to an independent effect of green areas on pregnancy outcomes, beyond those associated with lower pollution rates^40^. Associations between air pollution and prematurity in São Paulo have indeed been found by studies that considered micro-scale exposure^10^, as well as associations of air pollution with impairment of placental angiogenesis and reduced placental function^45^.

This study reinforces the importance of road afforestation for human health, especially in large cities. We found an inverse association between the number of street trees within each district, obtained using the Geosampa platform, and preterm birth. Interestingly, a study in New York City^44^, which is also a megacity, showed that the number of street trees—not the percentage of green areas in residential surroundings—and the access to large green or blue areas correlated beneficially with the rate of premature births^46^.

There is no consensus on the minimum amount of exposure to green areas and its beneficial effects on health. Urban planners have recommended the 3-30-300 rule, i.e. each resident should be able to see at least three trees from their home, school or workplace, have no less than 30% tree cover in their neighborhood and live within 300 m of a public green space^47^. In this study, we observed that the beneficial associations were found in the districts within the highest quartile of green areas. A previous study by our group, which assessed mental health in the São Paulo metropolitan region, showed similar results: the beneficial association that was found between anxiety and green areas was only significant in the last quartile^15^. In their UK study assessing mortality and morbidity, Mitchell et al.^48^ suggested that larger green areas may be more important for health effects than smaller spaces. When examining green areas by quartile, Tvina et al.^49^ also observed that the higher quartiles were associated with lower chances of preterm birth.

Kent et al.^50^ showed that, in the state of Alabama, United States, the frequency of premature births was higher among poorer African-American populations living in densely urbanized regions than among those living in rural areas. Our data reinforce these findings, showing that the chance of prematurity was higher in more densely built areas of São Paulo. Greater exposure to adverse environmental factors such as higher levels of air pollution, noise, temperature and stress may influence these results.

We found that few studies on this subject have been conducted outside the global north. It is, therefore, difficult to compare the results of the studies we found with our own, since the countries in which they were conducted differ greatly from Brazil. Castilo et al.^51^ noted the lack of data from middle- and low-income countries when studying health and green areas. One of the few studies found was carried out in Iran and only analyzed birth weight, not prematurity; in addition, this country’s climate is very different from that of São Paulo^52^.

This study has some limitations. The year chosen for analysis was 2012, as the database and orthophotos of the city of São Paulo were accessible for that year. It would be interesting to compare these data with the most recent ones. Another limitation is the use of satellite imagery to analyze air pollution, as it has a low spatial resolution and does not show much variation between the city’s districts. In the city of São Paulo in 2012, there was still no PM_2.5_ monitoring network with the broad spatial distribution required for this study. The access to green areas and their quality were also not assessed. Moreover, the irregular distribution and lack of proper management of dense green areas, in addition to the fear of violence while accessing them, certainly interfere with the potential they have to improve health in the city of São Paulo.

## CONCLUSIONS

Our data show that districts of São Paulo with more grass cover, street trees, and tree cover present a lower odds ratio for the occurrence of preterm births, which is reversed in more densely built areas. Additionally, the data reinforce the importance of intelligent urban planning: the city’s green areas need to be significantly densified based on strategies such as road afforestation.
