# Erratum

**DOI:** 10.11606/s1518-8787.2024058005504err

**Published:** 2024-04-25

In the article “Effects of land cover and air pollution on the risk of preterm births”, DOI https://doi.org/10.11606/s1518-8787.2024058005504, published on the Revista de Saúde Pública.2024;58:08, RSP corrects:

After references (page 14):

**Funding:** Fundação de Amparo à Pesquisa do Estado de São Paulo (Fapesp – Processes 16/15989-6; 09/02186-9; 09/53931-6 e 16/26082-1). Conselho Nacional de Desenvolvimento Científico e Tecnológico (CNPq - Process 304277/2019-3).

**Authors’ Contribution:** Study design and planning: TCLM, SRDMS, PHS, TM. Data collection, analysis, and interpreation: TCLM, JLP, WR, SRDMS, TM. Preparation or revision of the manuscript: TCLM, JLP, WR, PHS, DFSF, SRDMS, TM. Final version approval: TCLM, JLP, WR, PHS, DFSF, SRDMS, TM. Public responsibility for article content: TCLM, SRDMS, TM.

**Conflict of Interest:** The authors declare no conflict of interest.

