# Supplementary material for: Effects of land cover and air pollution on the risk of preterm births
Source: Rev Saude Publica. 2024 Mar 4;58:08. doi: 10.11606/s1518-8787.2024058005504 (PMC10926984; doi:10.11606/s1518-8787.2024058005504)
Supplement: Translation

# Translation: Efeitos da cobertura de solo e poluição do ar no risco de nascimentos prematuros

**Authors:** Tiana C. L. Moreira, Jefferson L. Polizel, Weeberb J. Réquia, Paulo Hilario Nascimento Saldiva, Demostenes F. da Silva Filho, Silvia Regina Dias Medici Saldiva, Thais Mauad

**Affiliations:** I Brasil Universidade de São Paulo. Faculdade de Medicina. Departamento de Patologia. São Paulo, SP, Brasil; II Brasil Escola Superior de Agricultura “Luiz de Queiroz”. Departamento de Ciências Florestais. Piracicaba, SP, Brasil; III Brasil Fundação Getúlio Vargas. Escola de Políticas Públicas e Governo. Brasília, DF, Brasil; IV Brasil Universidade de São Paulo. Faculdade de Medicina. Departamento de Obstetrícia e Ginecologia. São Paulo, SP, Brasil

**Keywords:** Parques Recreativos, Poluição do Ar, Recém-Nascido Prematuro, Ambiente Construído

## Abstract

**OBJETIVO:**

Avaliar a associação entre a idade gestacional e as áreas verdes, áreas construídas urbanas e a concentração de material particulado 2,5 (MP_2,5_) em São Paulo, analisando a distribuição irregular dessas áreas e os níveis de poluição acima do recomendado.

**MÉTODOS:**

A população utilizada no estudo foi a dos nascidos vivos no ano de 2012, com os dados do Sistema de Informações sobre Nascidos Vivo (Sinasc) na cidade de São Paulo. Por meio de imagens de satélites e realizando a classificação supervisionada, obtivemos a distribuição e quantidade de áreas verdes e de áreas construídas, na cidade de São Paulo, assim como as concentrações de MP_2,5_. Regressões logísticas foram utilizadas para obter possíveis associações.

**RESULTADOS:**

Os resultados do estudo mostram que menor percentual de áreas verdes está associado significativamente com maior chance de prematuridade. Maior densidade de construção foi associada positivamente com a razão de chance de nascimento prematuro. Não encontramos resultados significativos entre a poluição do ar (MP_2,5_) e prematuridade.

**CONCLUSÕES:**

Os resultados deste estudo demostraram que áreas mais verdes em relação às áreas menos verdes são menos associadas a nascimentos prematuros.

## INTRODUÇÃO

A associação entre desfechos gestacionais e exposições ambientais, como as áreas verdes urbanas e a poluição do ar, é um importante campo da epidemiologia ambiental.

Existem vários estudos demonstrando os benefícios de se morar próximo às áreas verdes e de frequentá-las em relação ao peso ao nascimento e/ou prematuridade^1^. Grazuleviciene et al.^4^realizaram um estudo com 3.416 mulheres no primeiro trimestre de gravidez, na Lituânia, investigando a influência benéfica da menor distância entre a residência e parques públicos e a pressão arterial sistêmica materna. Em outro estudo, gestantes que moravam até 1.250 m (ou 10 a 15 minutos de caminhada) de áreas verdes em cidades da Pensilvânia (EUA) apresentaram maior frequência de gestações a termo em relação a aquelas que moram a distâncias maiores do que 1.250 m^5^.

Grupos de menor poder aquisitivo parecem se beneficiar, especialmente, da exposição às áreas verdes. Na China, mães com menor poder aquisitivo expostas a essas áreas obtiveram os maiores benefícios dos desfechos de nascimento, principalmente relativos à prematuridade^6^. Akaraci et al.^1^, na Austrália, observaram que maiores coberturas de áreas verdes estavam relacionadas a menores chances de prematuridade na população mais vulnerável e com menor poder aquisitivo.

Outros estudos não encontraram evidências de que morar próximos a áreas verdes ou menos poluídas diminuía o risco de prematuridade. Asta et al.^7^, por exemplo, observaram que mulheres em Roma, mesmo morando mais próximas a áreas verdes, tinham aumentada probabilidade de prematuridade a cada aumento de 1˚C de temperatura, sem efeito modificador dos valores de MP_10_.

A exposição materna ao material particulado causou efeitos adversos sobre a prematuridade e peso ao nascer em diversos estudos ao redor do mundo^8^. Confirmando estes dados, um estudo realizado na cidade de São Paulo observou que um aumento de 10 μg/m^3^em O_3_e MP_10_estava associado à chance de prematuridade e baixo peso ao nascer^9^.

Nos anos de 2012 e 2013, foram registrados 348.337 nascidos vivos na cidade de São Paulo, com uma taxa de prematuridade de 11,9%, variando de 8,4% a 15,9% nos 96 distritos da cidade de São Paulo^10^, um valor muito semelhante às médias globais^11^. Leal et al.^12^observaram que, em uma população de 23.940 mulheres brasileiras em 2011 e 2012, 11,5% dos nascimentos foram prematuros; e que mães adolescentes, com baixa escolaridade e renda, formavam a maioria nessa taxa.

Existem poucos estudos em que variáveis ambientais e sua influência sobre desfechos gestacionais foram analisadas em São Paulo. A maior parte dos estudos foi realizada em países de maior poder aquisitivo que o nosso^8 , 13 , 14^ou em cidades com características distintas de poluição e de distribuição de áreas verdes das nossas megacidades. A distribuição das áreas verdes em São Paulo é bastante desigual, estando concentradas em locais mais ricos e intraurbanos. As áreas verdes localizadas em regiões periféricas são remanescentes das nativas, não localizadas na malha urbana e de pouco uso recreativo^15^. Os níveis de MP_2,5_na cidade de São Paulo, mensurados pelas estações da Companhia Ambiental do Estado de São Paulo (Cetesb), extrapolam os limites máximos estabelecidos pela Organização Mundial de Saúde (OMS)^18 , 19^.

Neste estudo, portanto, apresentamos dados analisando a exposição às áreas verdes e construídas e MP_2,5_materna, bem como a razão de chance de prematuridade no município de São Paulo no ano de 2012.

## MÉTODOS

### Área de Estudo

A cidade de São Paulo tem uma área de 1.521 km^2^e, em 2012, uma população de 11,37 milhões de habitantes^20^. O clima da cidade, de acordo com Koppen, é Cwa (clima subtropical úmido), caracterizado por um inverno seco e um verão chuvoso^21^.

A distribuição das áreas verdes na cidade de São Paulo é irregular, conforme demonstrado por Amato et al.^17^As maiores concentrações de áreas verdes estão em regiões de proteção ambiental, nas bordas da cidade.

### População de Estudo

A base de dados do Sistema de Informações sobre Nascidos Vivo (Sinasc), do ano de 2012, geocodificada pelo endereço completo das mães foi fornecida pela Coordenação de Epidemiologia e Informação da Secretaria Municipal de Saúde do Município de São Paulo ( Figura ), por meio de uma parceria para o desenvolvimento do projeto de pesquisa sobre Prematuridade no Município de São Paulo, aprovado pelo Comitê de Ética da Secretaria Municipal de Saúde (CAEE 26132714.1.0000.0086). Para este estudo, os endereços foram agrupados no nível de distritos da cidade. O Sinasc fornece variáveis como peso ao nascer, idade gestacional, tipo de gestação (única, dupla, tripla), presença de anomalias congênitas, tipo de parto, idade, escolaridade e ocupação da mãe e número de partos prévios (Portaria SVS nº 116/2009, de 11/02/2009).

**Figura:** População de estudo na cidade de São Paulo.

Neste estudo, foram analisados 174.215 registros dos nascidos vivos únicos. Para a análise, foram utilizadas as variáveis: peso ao nascer, idade gestacional, tipo de parto, data de nascimento, escala de Apgar 5, bem como idade, estado civil e escolaridade da gestante^22^. O banco de dados foi tratado mantendo apenas o peso acima de 0,5 e abaixo de 5 kg e excluindo linhas com dados em branco.

A idade da gestante foi categorizada em faixas, sendo elas: menor que 15; entre 15 e 19,9; entre 20 e 34,9; entre 35 e 39,9; e acima de 40 anos.

A variável escolaridade (última série concluída) foi utilizada conforme consta no banco de dados de Sinasc (nível de escolaridade), conforme descrito a seguir:

0. Sem escolaridade;

1. Fundamental I (1ª a 4ª série);

2. Fundamental II (5ª a 8ª série);

3. Médio (antigo 2º grau);

4. Superior incompleto;

5. Superior completo.

### Desfechos Gestacionais

Neste estudo a idade gestacional que o banco de dados do Sinasc fornece pode ser gerada tanto pelo dia da última menstruação quanto pelo ultrassom.

A idade gestacional é classificada de acordo com Guidelines for Perinatal Care (American Academy of Pediatrics) e The American College of Obstetricians and Gynecologists^23^:

Pré-termo extremo: menor que 28 semanas;

Muito pré-termo: 28 semanas a 31 semanas e 6 dias;

Pré-termo moderado: 32 semanas a 33 semanas e 6 dias;

Pré-termo tardio: 34 semanas a 36 semanas e 6 dias;

Pré-termo: menor que 37 semanas;

Termo inicial: 37 semanas a 38 semanas e 6 dias;

Termo total: 39 0/7 semanas a 40 semanas e 6 dias;

Termo tardio: 41 0/7 semanas a 41 semanas e 6 dias;

Pós-termo: 42 semanas ou mais.

Foi criada uma variável binária (0,1) referente à semana gestacional: quando prematuro é igual a 1 (menos de 37 semanas), e não prematuro igual a 0 (mais de 37 semanas).

A variável tipo de parto utilizada foi cesária ou parto normal.

O Apgar é um sistema de pontuação usado para avaliar rapidamente a saúde de um recém-nascido logo após o nascimento. Foi desenvolvido pela doutora Virginia Apgar, em 1952, e é composto por cinco categorias: frequência cardíaca, esforço respiratório, tônus muscular, reflexo de irritabilidade e cor da pele. Ele pode ser avaliado no primeiro minuto após o nascimento (Apgar 1), cinco minutos após o nascimento (Apgar 5) e, algumas vezes, após 10 minutos, quando o score for abaixo de 5. Neste estudo, iremos utilizar o Apgar 5^24 , 25^.

### Avaliação da Exposição e Cobertura do Solo

A área de exposição ao verde e de cobertura do solo utilizada foi analisada de acordo com a divisão administrativa do município em 96 distritos^26^.

O GeoSES foi utilizado como fator de correção socioambiental, que resume as principais dimensões do contexto socioeconômico brasileiro para fins de pesquisa^27^. O GeoSES é um índice composto, que resume as principais dimensões do contexto socioeconômico brasileiro para fins de pesquisa: educação, mobilidade, pobreza, privação social e material, renda, riqueza e segregação.

Os dados de poluição foram obtidos por meio de imagens de satélite obtidas do *The Copernicus Atmosphere Monitoring Service* (CAMS) de 2012. Extraímos os dados de material particulado 2,5 (MP_2,5_) em μg/m^3^das imagens, utilizando a média do período em cada distrito da cidade de São Paulo^28^. Neste estudo, utilizamos as médias anuais do poluente, que não levam em consideração as variações durante o ano, que dependem das estações e condições climáticas.

Neste estudo, usando o programa QGIS2.18.11, foram utilizados dois indicadores diferentes de exposição a áreas verdes: cobertura vegetal e o número de árvores de rua. Um mapa digital das localizações das árvores de rua em São Paulo no ano de 2010 foi fornecido pelo próprio município (Geosampa)^29^. O mapa identificou árvores urbanas em calçadas, ilhas de rua e rotatórias e excluiu árvores em praças, parques, reservas e áreas públicas e privadas internas. As imagens tinham uma resolução de 2 m em uma escala de 1:25.000.

As ortofotos utilizadas neste estudo foram cedidas pelo Instituto de Geociências e Cartografia do Estado de São Paulo e tinham uma resolução espacial de 2 m de lado do pixel, com três bandas espectrais: infravermelho próximo (NIR), azul e vermelho. A classificação da cobertura do solo foi realizada utilizando-se o algoritmo de Random Forest (RF) (programa QGIS2.18.11; Plugin Dtezaka). O RF é um poderoso algoritmo classificador de aprendizagem, bem como um dos métodos mais precisos de classificação da cobertura de solo^30^. Além disso, é um termo geral para métodos de conjunto que usam classificadores do tipo árvore para treinar o algoritmo, que cria várias árvores semelhantes a *Classification and Regression Tree* (CART)^31^.

Para classificação, para cada árvore na RF há uma votação para a classe mais popular (cor do pixel) na entrada de dados (polígono de amostras do treinador = entrada de dados). A saída do classificador é determinada pela maioria de votos da classe^32^. Para as amostras do treinador, a classificação das classes foi realizada de acordo com a cor do pixel e a assinatura espectral. Cento e cinquenta amostras de treinamento foram preparadas para cada classe de cobertura da terra. As imagens foram classificadas dentro das seguintes classes de cobertura do solo: copa das árvores, vegetação rasteira, solo nu, piso de cimento, piscina, sombra, telhado (branco, cinza, escuro, cerâmico), asfalto e rio/lago (adaptado de Myeong et al.^33^, 2003). Para a análise dos dados, considerou-se a soma das copas das árvores e da vegetação rasteira como espaço verde e a soma dos diferentes tipos de telhado como áreas construídas.

Usamos um esquema composto de falsa cor para permitir a detecção de vegetação na imagem. Neste tipo de representação, a vegetação aparece em diferentes tons de vermelho, dependendo do tipo e condição, devido à sua alta refletância na banda NIR^34^. Solo nu, estradas e edifícios podem aparecer em vários tons de azul, amarelo ou cinza, dependendo do material de composição. A composição ortofoto de falsa cor utilizada neste estudo foi R (canal 1) = banda NIR, G (canal 2) = banda vermelha, B (canal 3) = banda azul.

A acurácia da classificação foi determinada por meio de uma matriz de erro de classificação, o índice Kappa. Os mapas temáticos utilizados neste estudo apresentaram valores de Kappa iguais ou superiores a 81%, o que é considerado uma classificação acurada de acordo com Landis e Koch^35^.

Bancos de dados de árvores de rua e cobertura do solo foram avaliados nos 96 distritos.

### Análise de Dados

Estas análises foram realizadas para entender a associação entre a semana gestacional e as variáveis de exposição ambiental e sociodemográficas. Examinamos cada variável em modelos sem ajuste, que, a seguir, foram ajustados para modelos logísticos, nos quais as variáveis mostraram associação com, pelo menos, um dos desfechos na análise. A regressão logística binária foi utilizada para as variáveis dependentes dicotômicas. O nível de significância adotado foi de p = 0,05. Os resultados das regressões logísticas foram apresentados como *odds ratio* (intervalos de confiança de 95%).

Os modelos logísticos foram controlados para a raça, idade, escolaridade e estado civil, poluição do ar e condição socioeconômica.

A cobertura do solo foi utilizada em quartis e dividida da seguinte forma ( Tabela 2 ):

**Tabela 2:** Distribuição dos quartis das coberturas de solo e material particulado (MP)2,5.

Cobertura de solo	Q1 (%)	Q2 (%)	Q3 (%)	Q4 (%)
Áreas verdes	15,4 (24,8)	25,8 (33,6)	34,6 (45,0)	46,0 (84,8)
Cobertura arbórea	6,9 (14,7)	15,7 (23,3)	24,3 (33,6)	34,6 (73,4)
Vegetação rasteira	3,4 (8,0)	9,05 (9,31)	10,31 (11,6)	12,6 (17,7)
Área construída	4,6 (21,0)	22,0 (29,9)	30,9 (36,7)	37,7 (49,7)
MP_2,5_	11,8 (12,1)	13,2 (12,3)	13,3 (12,7)	13,7 (13,7)
Árvores de rua	0,5 (4,8)	5,82 (6,8)	7,8 (9,73)	10,73 (16,2)

- Q1 – a menor percentagem de cobertura do solo dentro de 25% da população;
- Q2 – a segunda menor percentagem de cobertura do solo dentro de 25% da população (até à mediana);
- Q3 – a segunda maior percentagem de cobertura do solo dentro de 25% da população (acima da mediana);
- Q4 – a maior percentagem de cobertura do solo dentro de 25% da população.

Todos os modelos foram construídos separando cada variável de cobertura do solo, para evitar a multicolinearidade. Uma nova variável, chamada “área verde”, foi criada, definida como a soma de árvores e de vegetação rasteira; outra, chamada “área construída”, foi a soma de todos os telhados de cores diferentes. O nível de significância adotado foi de p < 0,05.

## RESULTADOS

Dos 174.215 registros de nascidos únicos vivos, foram retirados os casos cujos endereços eram de fora do município de São Paulo e os que não continham as informações relativas às variáveis utilizadas no estudo, resultando em 166.384 registros, conforme observado na Tabela 1 .

**Tabela 1:** Análise descritiva das variáveis de nascimento (1a - sociodemográfico e 1b- saúde recém-nascido).

Variáveis	n	%
Sociodemográfico (n = 166.384)		
Idade da mãe		
Média (CV%)	27,7 (23,8%)	
Mediana [Min, Max]	28,0 [11,0, 52,0]	
Desvio padrão	6,6	
Estado civil mãe		
Solteira	75.261	43,2
Casada	68.082	39,08
Viúva	278	0,16
Separada/divorciada	2.451	1,41
União estável	27.878	16
Sem dados	58	0,03
Escolaridade		
Sem escolaridade	17	0,01
Fundamental 1	294	0,17
Fundamental 2	2.019	1,16
Médio	20.487	11,76
Superior incompleto	103.185	59,23
Superior completo	47.896	27,49
Sem dados	169	0,1
Saúde recém-nascido (n = 166.384)		
Escala de Apgar		
0		0,09
1		0,13
2		0,05
3		0,06
4		0,11
5		0,22
6		0,33
7		0,94
8		4,23
9		34,86
10		58,65
Sem dados		0,1
Presença de anormalidade congênitas		
Presente		1,84
Ausente		98,02
Sem dados		0,1
Tempo gestacional		
Pré termo	18.434	12,31
Termo inicial	61.147	36,06
Termo total	73.537	42,63
Termo tardio	9.702	5,62
Pós termo	3.628	2,12
Peso nascimento (CV)		
Média (CV)	3.162,90	17,7
Mediana [Min, Max]	[500, 4.990]	
Desvio padrão	354	
Sem dados	6	0
Idade gestacional do parto (CV)		
Média (CV%)	38,3	5,8
Mediana [Min, Max]	39,0 [19,0, 46,0]	
Desvio padrão	2,21	
Sem dados	2.196	1,3

Na Tabela 2 , podemos observar a distribuição dos quartis de acordo com as coberturas de solo, mostrando que “área verde” possui uma variação que vai de 15,4 % até 84,8%, já a poluição do ar varia muito pouco, de 11.8 até 13.7 μg/m^3^.

Os resultados da análise “modelos sem ajuste e logísticas” podem ser vistos na Tabela 3 . Os estados civis “solteira” e “união estável” mostraram associação (aumento de 2% e 4%, respectivamente) com a prematuridade, em relação ao estado civil “casada”, nas análises modelo logístico sem ajuste e mantendo a significância na análise logística. A faixa etária na análise modelo logístico sem ajuste foi significante em todas as faixas etárias, em relação à faixa etária de 20 até 35 anos. Na análise logística, apenas as faixas acima de 35 anos mostraram estar associadas com a prematuridade. A média GeoSES está associada positivamente com a prematuridade nas regressões modelo logístico sem ajuste apesar de marginal, porém, essa associação não ocorreu na análise logística.

**Tabela 3:** Resultado dos modelos sem ajuste e modelos logísticos para idade gestacional do parto < 37 semanas e cobertura de solo.

Idade Gestacional do Parto		Sem ajuste			Logística		
	Preditor	Razão de chances	IC	p	Razão de chances	IC	p
Estado civil - ref solteira	Casada ref	---------	------------	------	-------	-------------	------
	Solteira	1,11	1,08 – 1,15	**0,001**	1,12	1,04 – 1,12	**0,001**
	Viúva	1,09	0,73 – 1,56	0,629	1,10	0,71– 1,52	0,747
	Separada/divorciada	1,06	0,92 – 1,20	0,379	1,06	0,92 – 1,20	0,418
	união estável	1,11	1,06 – 1,16	**0,001**	1,11	1,02 – 1,12	**0,003**
Escolaridade - ref sem escolaridade	Sem escolaridade	1,07	0,63 – 1,80	0,794	1,06	0,60 – 1,73	0,815
	Fundamental 1	1,46	1,04 – 2,05	**0,026**	1,42	1,00– 1,97	**0,040**
	fundamental 2	1,27	1,10 – 1,45	**0,001**	1,23	1,07– 1,41	**0,003**
	médio	1,20	1,14 – 1,26	**0,001**	1,17	1,10 – 1,23	**0,001**
	superior incompleto	1,09	1,04 – 1,12	**0,001**	1,06	1,02 – 1,10	**0,003**
	superior completo ref	---------	------------	------	-------	-------------	------
Idade mãe - ref 20-34,9	Ref : 20-34,9	---------	------------	------	-------	-------------	------
	=15	1,59	1,30 – 1,92	**0,001**	1,08	0,86 – 1,36	0,472
	15-19,9	1,27	1,21 – 1,32	**0,001**	1,05	0,99 – 1,11	0,064
	35-39,9	1,19	1,13 – 1,23	**0,001**	1,20	1,14 – 1,27	**0,001**
	>=40	1,43	1,32 – 1,54	**0,001**	1,30	1,18 – 1,42	**0,001**
Raça - ref branca	Branca	---------	------------	------	-------	-------------	------
	Negra	1,09	1,02 – 1,15	**0,006**	1,04	‘	0,142
	Amarela	0,93	0,80 – 1,06	0,278	0,94	0,82 – 1,08	0,426
	Parda	1,01	0,97 – 1,04	0,519	0,96	0,93 – 1,00	0,065
	Indígenas	0,86	0,68 – 1,06	0,177	0,80	0,63 – 0,99	**0,050**
Tipo de parto - ref normal	Parto normal	---------	------------	------	-------	-------------	------
	Parto cesárea	0,98	0,94 – 1,00	0,145	0,96	0,92 – 0,99	**0,039**
Consulta pré-natal	Número de consultas	0,933	0,92- 0,93	**0,001**	0.93	0.93 - 0.94	**0.022**
Dados nascimento	APGAR aos 5 minutos	0,82	0,81 – 0,83	0,001	0,97	0,96 – 0,98	**0,001**
	Peso ao nascimento (kg)	1,00	0,99 – 0,99	**0,001**	0,99	0,99 – 0,99	**0,001**
Espaços verdes - quartis	Área verde 1 Q [15,4-24,8]	1,07	1,02 – 1,11	**0,002**	1,08	1,03 - 1,13	**0,001**
	Área verde 2 Q [25,8-33,6]	1,04	0,99 – 1,08	0,104	1,05	1,00 – 1,10	**0,018**
	Área verde 3 Q [34,6-45]	0,99	0,94 – 1,029	0,516	0,99	0,94 - 1,3	0,671
	Área verde 4 Q [46-84,8]	---------	------------	------	-------	-------------	------
Cobertura arbórea - quartis	Cobertura arbórea 1 Q [6,9-14,7]	1,06	1,01 – 1,10	**0,011**	1,07	1.02- 1.12	**0,001**
	Cobertura arbórea 2 Q [15,7-23,3]	1,03	0,98 – 1,07	0,206	1,04	0,99-1,09	0,059
	Cobertura arbórea 3 Q [24,3-33,6]	0,98	0,93 – 1,02	0,384	0,98	0,94 – 1,03	0,571
	Cobertura arbórea 4 Q [34,6-73,4]	-------	-------------	------	-------	-------------	------
Vegetação rasteiro - quartis	Verde rasteiro - 1 Q [3,4-8]	1,11	1,06 – 1,16	**0,001**	1,09	1,04 – 1,14	**0,001**
	Verde rasteiro - 2 Q [9,05-9,31]	1,11	1,06 – 1,16	**0,001**	1,08	1,03 – 1,13	**0,001**
	Verde rasteiro - 3 Q [10,31-11,6]	1,04	0,99 – 1,08	0,125	1,02	0,97 – 1,07	0,301
	Verde rasteiro - 4 Q [12,6-17,7]	-------	-------------	------	-------	-------------	------
Área construída - quartis	área construída - 1 Q [4,6-21]	---------	------------	------	-------	-------------	------
	área construída - 2 Q [22-29,9]	1,02	0,97 – 1,06	0,297	1,03	0,99 – 1,08	0,123
	área construída - 3 Q [30,9-36,7]	1,02	0,97 – 1,06	0,314	1,02	0,97 – 1,07	0,357
	área construída - 4 Q [37,7-49,7]	1,09	1,04 – 1,13	**0,001**	1,09	1,04 – 1,14	**0,001**
Pm_2,5_- quartis	PM_2,5_- 1 Q[11,8-12,1]	---------	------------	------	-------	-------------	------
	PM_2,5_- 2 Q[13,2-12,3]	0,98	0,93 – 1,01	0,275	0,97	0,93 – 1,02	0,380
	PM_2,5_- 3 Q [13,3-12,7]	1,02	0,97 – 1,06	0,388	1,01	0,96 – 1,05	0,613
	PM_2,5_- 4 Q [13,7-13,7]	0,99	0,94 – 1,03	0,546	1,00	0,95 – 1,05	0,943
	MediaGeoSES	0,96	0,91 – 0,99	**0,035**	1,00	0,95 – 1,05	0,915
Árvores de rua – quartis (Ref: 9,73-16,2)	Árvores de rua - 1 Q [0.53-4.82]	1,01	0,96 – 1,05	0,595	1.00	0.95-1.04	0.940
	Árvores de rua - 2 Q [5,82-6,8]	0,97	0,92 – 1,00	0,11	0.95	0.91-0.99	**0.034**
	Árvores de rua - 3 Q [7,8-9,73]	1,00	0,95 – 1,04	0,975	1.01	0.95-1.04	0.945
	Árvores de rua - 4 Q [9,73-16,2]	-------	-------------	------	-------	-------------	------

MP: material particulado; IC95%: intervalo de confiança de 95%.

Na análise de regressão logística ( Tabela 3 ), a prematuridade se associou ao estado civil “solteiro” (OR = 1,12; IC95% 1,04–1,12; p = 0,001) e “união estável” (OR = 1,1; IC95% 1,02–1,12; p = 0,003), em relação ao estado civil “casado”; presença de árvores de rua no segundo quartil (Q2: OR = 0,95; IC95% 0,91–0,99; p = 0,034) dentro dos diferentes distritos, em relação ao quarto quartil, mostrou mais chance de prematuridade. A idade da mãe ser maior de 35 anos ou de 35 a 39,9 anos: OR = 1,20; IC95% 1,14–1,27; p = 0,001; e acima de 40 anos: OR = 1,30; IC95% 1,18–1,4; p = 0,001, indicou mais chance de prematuridade em relação a faixa etária de 20 até 34,9 anos. A porcentagem de áreas construídas no quarto quartil (OR = 1,09; IC95% 1,04–1,14; p = 0,001), dentro de cada distrito, mostrou aumento da associação com prematuridade em relação ao primeiro quartil. A porcentagem total de áreas verdes (Q1: OR = 1,08; IC95% 1,03–1,13; p = 0,001 e Q2: OR = 1,05; IC95% 1,00–1,10; p = 0,018), cobertura arbórea (Q1: OR = 1,07; IC95% 1,02–1,12; p = 0,001) e vegetação rasteira (Q1: OR = 1,09; IC95% 1,04–1,14; p = 0,001 e Q2: OR = 0,91; IC95% 1,00–1,10; p = 0,018) mostraram associação com prematuridade e mais chances de nascimentos prematuros, em relação ao quarto quartil de cada uma das respectivas coberturas de solo. O número de consultas pré-natal também mostrou associação com os nascimentos prematuros.

Não foram encontradas associações estatísticas entre a concentração de MP_2,5_nos distritos e número de nascidos prematuros.

## DISCUSSÃO

Neste estudo, analisamos a influência do tipo de cobertura do solo sobre a frequência de prematuridade na cidade de São Paulo, em 2012. Observamos que a porcentagem dos diversos tipos de áreas verdes nos distritos da cidade pôde diminuir de 5% até 9% a chance de nascimentos prematuros. Os resultados deste estudo também mostraram que um menor percentual de áreas verdes está associado significativamente a maior chance de prematuridade, assim como áreas densamente construídas (> 37,7% de área construída neste estudo). Não houve influência dos níveis de MP_2,5_nos parâmetros estudados. De nosso conhecimento, este é o primeiro estudo mostrando as associações benéficas entre a existência de áreas verdes urbanas e a baixa da prematuridade no Brasil.

A prematuridade também está relacionada a outros fatores, como raça, estado civil, idade materna, tipo de parto, consulta pré-natal, peso e Apgar, já bem estabelecidos na literatura^36^. Neste estudo, observamos que indígenas têm uma maior chance de prematuridade, assim como descrito por Martinelli et al.^36^e Barreto et al.^37^

O estado civil e a idade da mãe também influenciam na prematuridade. Neste estudo, gestantes com idade acima de 35 anos, solteiras ou em união estável possuem mais chance de parto prematuro, estes achados corroboram com resultados de outros estudos^38^.

O aumento de consultas pré-natal também está bem estabelecido na literatura como fator de menor risco de prematuridade^36 , 39^. Este fator também foi observado neste estudo.

Muitos estudos já foram realizados mostrando a associação benéfica entre exposição a áreas verdes e desfechos gestacionais, mas grande parte foi realizada em países de maior renda ou Índice de Desenvolvimento Humano^40^. Meta-análises revelam que tais associações são mantidas quando há alta porcentagem de área verde residencial.

Villeuneuve et al.^41^observaram que a porcentagem de área verde residencial esteve associada positivamente à redução dos riscos de nascimento prematuro, baixo peso ao nascer e nascituros pequenos para a idade gestacional; diferentemente deste estudo, em que a única associação positiva foi a prematuridade. Estudos que associam status socioeconômico a maiores benefícios das áreas verdes apontam que os efeitos benéficos são maiores nas populações desprovidas^42^, com exceção de um estudo australiano que mostrou benefícios desproporcionais entre mulheres de bairros mais afluentes^43^. Nesta pesquisa, usamos o índice GeoSES dentro de cada distrito, mas associações significativas entre este índice e prematuridade nos diferentes distritos não foram encontradas na análise logística. Na cidade de São Paulo, a maior ocorrência de áreas verdes não está necessariamente ligada aos índices socioeconômicos. A região sul da cidade, por exemplo, tem grandes áreas de cobertura vegetal nativa em distritos com baixos índices socioeconômicos. Já na região oeste, os maiores índices de cobertura vegetal estão nas áreas mais afluentes da cidade. Mesmo que, provavelmente, a qualidade e o acesso a estas áreas verdes sejam diferentes nas duas regiões.

Os mecanismos específicos pelos quais as áreas verdes trariam benefícios gestacionais não são conhecidos, mas algumas vias são plausíveis: 1) efeito psicológico restaurativo na mãe, com diminuição do estresse; 2) ação direta na saúde cardiovascular, por aumentar atividade física, e na saúde mental, por estimular a coesão social; 3) ação indireta por melhora das condições ambientais de poluição, temperatura e umidade^43^.

As áreas verdes são capazes de melhorar o microclima de regiões e diminuir a poluição^2^. Porém, assim como Akaraci et al.^40^em um estudo em Sydney, Austrália, não encontramos associação entre MP_2,5_e prematuridade, diferentemente de estudos em outros países^40 , 43 , 44^. Pelo fato de as medidas de poluição neste estudo terem sido derivadas de imagens de satélite, é possível que diferenças de exposição individuais não tenham sido captadas. Além disso, este resultado aponta um efeito independente das áreas verdes sobre os resultados do nascimento, para além dos associados a menores taxas de poluição^40^. De fato, associações entre poluição do ar e prematuridade em São Paulo foram encontrados em estudos onde houve microescala de exposição^10^e, também, prejuízo na angiogênese placentária e redução da função placentária^45^.

Este estudo reforça a importância da arborização viária na saúde humana, principalmente nas grandes cidades. Encontramos associação inversa entre o número de árvores de rua dentro de cada distrito, obtidas por meio da plataforma Geosampa, e os nascimentos prematuros. Curiosamente, um estudo na cidade de Nova Iorque^44^, também uma megacidade, mostrou que o número de árvores de ruas – e não a porcentagem de áreas verdes no entorno residencial – e o acesso a grandes áreas verdes ou azuis correlacionaram-se de maneira benéfica com o índice de nascimentos prematuros^46^.

Não existe consenso sobre a quantidade mínima de exposição às áreas verdes e seus efeitos benéficos à saúde. Urbanistas têm recomendado a regra do 3-30-300, isto é, que cada morador tenha pelo menos a vista de três árvores de sua casa, escola ou trabalho, não menos de 30% de cobertura arbórea no seu bairro e que esteja a 300 m de um espaço público de áreas verdes^47^. Neste estudo, observamos que as associações benéficas se apresentam nos distritos dentro do maior quartil de áreas verdes. Estudo anterior do nosso grupo, avaliando a saúde mental na região metropolitana de São Paulo, mostrou resultados similares, em que a associação benéfica entre ansiedade e áreas verdes só se mostrou significante no último quartil^15^. Mitchell et al.^48^sugerem que maiores áreas verdes podem ser mais importantes para os efeitos na saúde do que espaços menores, em seu estudo no Reino Unido que avalia mortalidade e morbidade. Tvina et al.^49^também observaram que, examinando as áreas verdes por quartil, os maiores foram associados a menores chances de parto prematuro.

Kent et al.^50^demonstraram que, no estado do Alabama, havia maior frequência de nascimentos prematuros nas populações afro-americanas mais pobres que viviam em regiões densamente urbanizadas, quando comparadas com a região rural. Nossos dados reforçam esses achados, mostrando maior chance de prematuridade em regiões mais densamente construídas de São Paulo. Maior exposição a fatores ambientais adversos, como maiores níveis de poluição do ar, ruído, temperatura e estresse podem influenciar estes resultados.

Poucos estudos são encontrados fora do eixo do norte global, sendo difícil comparar resultados de países com tantas diferenças, como o Brasil. Castilo et al.^51^observaram a falta de dados de países de média e baixa renda estudando saúde e áreas verdes. Um dos poucos estudos encontrados foi desenvolvido no Irã e analisou apenas peso ao nascer e não prematuridade, além disso, o clima do país é muito diferente do de São Paulo^52^.

Este trabalho possui algumas limitações. O ano de estudo escolhido foi 2012, pois nele havia acessibilidade ao banco de dados e as ortofotografias da cidade de São Paulo. Seria interessante comparar estes dados com os mais recentes. Outra limitação é a utilização da imagem de satélite para a análise de poluição do ar, que possui uma baixa resolução espacial, não mostrando uma grande variação entre os distritos da cidade. Na cidade de São Paulo, em 2012, ainda não existia a rede de monitoramento de MP_2,5_com a ampla distribuição espacial necessária para este estudo. Também não foi avaliado o acesso às áreas verdes, nem sua qualidade. Além disso, a distribuição irregular, a falta de manejo adequado e o medo da violência ao acesso a áreas verdes densas certamente interferem no potencial salutogênico das áreas verdes na cidade de São Paulo.

## CONCLUSÕES

Nossos dados apontam que nos distritos de São Paulo com maior cobertura arbórea, vegetação rasteira e número de árvores de rua existe uma razão de chance menor de nascimentos prematuros, que inverte-se em áreas mais densamente construídas. Além disso, é reforçada a necessidade de planejamento inteligente, no sentido de adensar significativamente as áreas verdes da cidade, incluindo arborização urbana.
