# Supplementary material for: Erratum
Source: Rev Saude Publica. 2024 Apr 25;58:08err. doi: 10.11606/s1518-8787.2024058005504err (PMC11090609; doi:10.11606/s1518-8787.2024058005504err)
Supplement: Translation

# Translation: Errata

No artigo “Efeitos da cobertura de solo e poluição do ar no risco de nascimentos prematuros”, DOI https://doi.org/10.11606/s1518-8787.2024058005504, publicado na Revista de Saúde Pública.2024;58:08,a RSP corrige:

Após as referências (página 14):

**Financiamento:** Fundação de Amparo à Pesquisa do Estado de São Paulo (Fapesp – processos 16/15989-6; 09/02186-9; 09/53931-6 e 16/26082-1). Conselho Nacional de Desenvolvimento Científico e Tecnológico (CNPq - processo 304277/2019-3).

**Contribuição dos Autores:** Concepção e planejamento do estudo: TCLM, SRDMS, PHS, TM. Coleta, análise e interpretação dos dados: TCLM, JLP, WR, SRDMS, TM. Elaboração ou revisão do manuscrito: TCLM, JLP, WR, PHS, DFSF, SRDMS, TM. Aprovação da versão final: TCLM, JLP, WR, PHS, DFSF, SRDMS, TM. Responsabilidade pública pelo conteúdo do artigo: TCLM, SRDMS, TM.

**Conflito de Interesses:** Os autores declaram não haver conflito de interesses.
